# Forefoot Function after Hallux Valgus Surgery: A Systematic Review and Meta-Analysis on Plantar Load Measurement

**DOI:** 10.3390/jcm12041384

**Published:** 2023-02-09

**Authors:** Duo Wai-Chi Wong, James Chung-Wai Cheung, Jia-Guo Zhao, Ming Ni, Zu-Yao Yang

**Affiliations:** 1 Jockey Club School of Public Health and Primary Care, Faculty of Medicine, The Chinese University of Hong Kong, Hong Kong 999077, China; 2 Department of Biomedical Engineering, Faculty of Engineering, The Hong Kong Polytechnic University, Hong Kong 999077, China; 3 Department of Orthopaedic Surgery, Tianjin Hospital, Tianjin 300211, China; 4 Department of Science Development, Ruijin Hospital, Shanghai Jiao Tong University, Shanghai 200240, China; 5 Department of Orthopaedics, Pudong New Area People’s Hospital, Shanghai 201299, China

**Keywords:** bunion, hallux abducto valgus, metatarsus primus varus, pedobarography, postoperative evaluation

## Abstract

While hallux valgus (HV) surgeries are useful for correcting skeletal alignment problems, their effects on plantar load, which reflects forefoot functions, are less understood. The objective of this study is to conduct a systematic review and meta-analysis on the plantar load change after HV surgeries. A systematic search of Web of Science, Scopus, PubMed, CENTRAL, EMBASE, and CINAHL was performed. Studies that assessed the pre- and post-operative plantar pressure of HV patients undergoing surgeries and reported load-related parameters over the hallux, medial metatarsal, and/or central metatarsal regions were included. Studies were appraised by using the modified NIH quality assessment tool for before-after study. Studies suitable for meta-analysis were pooled with the random-effects model, using the standardized mean difference of the before-after parameters as an effect measure. Twenty-six studies containing 857 HV patients and 973 feet were included for the systematic review. Meta-analysis was conducted on 20 of them, and most studies did not favor HV surgeries. Overall, HV surgeries reduced the plantar load over the hallux region (SMD −0.71, 95% CI, −1.15 to −0.26), indicating that forefoot function worsened after surgeries. For the other five outcomes, the overall estimates were not statistically significant, indicating that surgeries did not improve them either. There was substantial heterogeneity among the studies, which in most cases could not be resolved by pre-planned subgroup analyses by surgical classification, year of publication, median age of patients, and length of follow-up. Sensitivity analysis removing lower-quality studies showed that the load integrals (impulse) over the central metatarsal region significantly increased (SMD 0.27, 95% CI, 0 to 0.53), indicating that surgeries increased the risk of transfer metatarsalgia. There is no solid evidence that HV surgeries could improve forefoot functions from a biomechanical point perspective. Currently available evidence even suggests that surgeries might reduce the plantar load over the hallux and adversely affect push-off function. The reasons behind and the effectiveness of alternative surgical methods warrant further investigation.

## 1. Introduction

Hallux valgus (HV) is one of the most prevalent foot problems, affecting nearly one-third of the female population and exacerbating with increasing age [1]. It is characterized by medial deviation of the first metatarsal, lateral deviation of the hallux, and a swollen medial eminence (bunion). HV not only causes pain and difficulty in fitting shoes [2,3], but also attenuates muscle activity and gait stability, which foists risks of falls, knee injuries, and ankle sprains [4,5]. In severe cases, subluxation of the metatarsophalangeal joint and dislocation of sesamoids may happen. In addition, HV could be associated with secondary deformities of the lesser toes [6]. Corrective surgeries are often recommended to patients with severe angular deformity (HV angle > 40° and intermetatarsal angle > 20°), particularly those with serious incongruent and hypermobile joint conditions [7]. The correction methods could be broadly classified as osteotomy, arthrodesis, arthroplasty, and soft tissue procedures. Though osteotomy is the most common option [8], the choice of surgical method could depend on the multiplicity of patient condition and surgeon preference [9]. Osteotomy could be conducted in a minimally invasive or percutaneous way [10]. Percutaneous surgery is carried out through the skin, while minimally incision surgery could be a procedure with an exposure level between open surgery and percutaneous surgery [11]. Nevertheless, these techniques are currently recommended for mild cases and require a high degree of expertise in arthroscopic and endoscopic surgery [10,11].

Radiographic examination is the gold standard to evaluate surgical outcomes since the primary goal of surgery is to correct skeletal alignment [12]. However, X-ray-proven bone realignment may not reflect sufficient restoration of foot functions. Clinical assessment exploits patient-reported outcomes on the perceived pain and function postoperatively, in which the American Orthopaedic Foot and Ankle Society (AOFAS) scales are the most widely utilized instrument [13], especially the AOFAS hallux metatarsophalangeal-interphalangeal scale (Hx-MTP-IP). The AOFAS Hx-MTP-IP scale is a 100-point grading scale with 40 points to evaluate pain level and 15 points to evaluate hallux alignment [13]. The remaining 45 points are used to assess functions, including limitations in daily activity, footwear requirements, joint range of motion, and stability [13]. Nevertheless, it was argued that the AOFAS scales lack precision and have not been appropriately validated [14,15].

**Figure 1:**
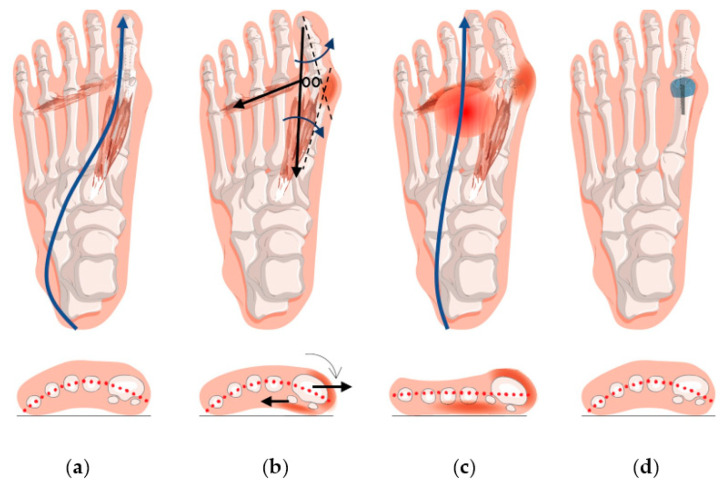
Pathomechanism of HV and transfer metatarsalgia: (**a**) The first ray plays an important role in push-off functions. The blue line illustrates the center of pressure trajectory during gait; (**b**) since the first metatarsal is less stable without any muscle insertions, it might deviate medially, conforming with the direction of the push-off load. The black arrows represent the directions of muscle force. The blue arrows represent the deformity directions of the bones [7]. The hallux and the sesamoids are hold in position by the muscle and plantar aponeurosis. Therefore, the hallux looks laterally deviated relative to the first metatarsal, while the sesamoids beneath the first metatarsal head are gradually exposed and disanchored. Plantarflexors, abductors, and stabilizing muscles of the first ray would become lateral to the longitudinal axis of the first ray and distribute deforming forces [7]. The problem aggravates with higher exposure and loading of the forefoot, such as wearing high-heeled shoes [26]; (**c**) when the muscles and the sesamoids that served as the fulcrum are deranged, the load-carrying capability and push-off functions are compromised and compensated by other forefoot regions, resulting in transfer metatarsalgia. The blue line represents the lateralized center of pressure trajectory; (**d**) the bone alignment and sesamoid positions shall be corrected after HV surgeries, but whether the center or pressure trajectory and thus load-carrying capability of the first ray could be restored is in doubt.

On the other hand, biomechanical evaluations targeting foot loading and functions have emerged recently, the results of which implicate risks of complications, recurrence, and walking instability [12,16,17]. Biomechanically, the failure in medial forefoot functions induced by HV transfers the load to the central metatarsal regions, causing transfer metatarsalgia [18]. The assessment of postoperative forefoot function is therefore imperative. Plantar pressure measurement (pedobarography), which quantifies the biomechanics of plantar foot functions, has been proven to be a reliable clinical tool to evaluate interventions [19,20,21]. Transfer metatarsalgia (midfoot pain) is caused by the aberrant high load that is shifted from the failed medial forefoot [22]. Therefore, an increase in the medial forefoot pressure and a reduction in the central forefoot pressure (i.e., medialized pressure) indicate the restoration of push-off function and pain relief. There is strong evidence that HV individuals had lower medial forefoot but higher central forefoot loading [22,23]. The pathomechanism of HV and transfer metatarsalgia is illustrated in Figure 1. However, previous studies evaluating the plantar pressure after surgeries reported inconsistent results. For example, Lorei et al. [24] reported that medial forefoot pressure increased after surgery, while Brodsky et al. [25] reported that it did not change notably. It was unclear whether the discrepancy was due to small sample sizes (and consequent insufficient statistical power) or other reasons.

The objective of this study is to conduct a systematic review and meta-analysis of studies that evaluated the changes in plantar load outcomes of medial and central forefoot after HV surgeries. We hypothesized that (1) surgery increases plantar load on the hallux and medial metatarsal region; (2) surgery decreases plantar load on the central metatarsal region.

## 2. Materials and Methods

### 2.1. Subsection Literature Search and Study Selection

This systematic review was conducted according to the Preferred Reporting Items for Systematic Reviews and Meta-analyses (PRISMA) guideline [27]. A literature search was performed on 3 October 2021, in Web of Science, Scopus, PubMed, CENTRAL, EMBASE, and Cumulated Index to Nursing and Allied Health Literature (CINAHL) using combinations of relevant key terms related to HV, surgery, and plantar pressure measurement. Details on search terms and strategy are available in Table S1. Studies that assessed the pre- and post-operative plantar pressure over the segmented region of HV patients undergoing surgical interventions and reported at least one load-related parameter over the hallux, medial metatarsal, or central metatarsal regions, regardless of the original study design and the number of arms within the study, were included in this review. Load was a collective class of parameters that included (maximum or average) pressure, force, and their time integrals (i.e., impulse). Studies were excluded if they involved patients with rheumatoid arthritis, diabetic foot, or re-surgery. Cadaveric, sawbone, computer simulations, and pure theoretical/mathematical research were also excluded. An additional search was performed by manually checking the reference lists of eligible articles. Literature search and screening were independently conducted by the first two authors. Data were extracted by the first author. Any disagreements were resolved by seeking consensus with the corresponding author. All references were exported to EndNote software. The protocol of the systematic review was registered in PROSPERO (CRD42021264693).

### 2.2. Data Extraction

The basic information of the eligible studies, including sample size (subject and feet), age, surgical procedure, duration of follow-up, outcome parameters, and main numerical results were extracted. If a study only provided charts or graphs without the numeric values needed for meta-analysis, the numeric values were estimated using GetData Graph Digitizer (http://getdata-graph-digitizer.com, accessed on 20 January 2023) by normalizing the lengths of the chart bars to the scales of the chart axes. For each outcome, the standardized mean difference (*β*) and its standard error (*s_β_*) were estimated by Equations (1) and (2) below when relevant data were available, according to the Cochrane guidelines [28].

(1) $$\hat{\beta }=\frac{\mu_{post-pre}}{\sqrt{\frac{\sigma_{pre}^{2}+\sigma_{post}^{2}}{2}}}$$

(2) $$s_{\hat{\beta }}=\sqrt{\frac{1}{N}+\frac{{\hat{\beta }}^{2}}{2N}}\times \sqrt{2\left(1-R\right)}$$

where *μ_post-pre_*, *σ_pre_*, *σ_post_*, *N*, and *R* represent the before-after mean difference, the standard deviation of pre-operative mean, the standard deviation of post-operative mean, the sample size, and the correlation coefficient, respectively, with the *R* assumed to be 0.5.

### 2.3. Methodological Quality Assessment

We conducted methodological quality assessment using a modified National Institute of Health (NIH) quality assessment tool for the before-after study [29]. The original NIH tool consisted of 12 yes/no items plus one question on overall quality rating. We removed the multilevel (e.g., hospital) effects, sample size, and statistical analysis items since they did not affect the quality of the data included for our analysis. Moreover, we discarded the overall quality rating item because the guidelines mentioned that there were no specific rules for deriving an overall rating, and that it was too subjective. The revised instrument had a total of 9 items (Table S2).

### 2.4. Statistical Analysis

We conducted meta-analyses on two outcomes, i.e., load and impulse (time-integral of load), over the hallux, medial metatarsal regions, and central metatarsal regions, respectively, giving six combinations, or in other words, six meta-analyses. The meta-analyses were conducted by the first author (D.W.-C.W.) and verified by the corresponding author (Z.-Y.Y.). The load-related outcomes included maximum or average force, pressure, or load. The impulse-related outcomes included pressure time-integral and force time-integral. If a paper reported both the maximum and average value, we selected the average value for analysis. If a paper reported both force and pressure, we selected the pressure parameter for analysis. For each outcome, a standardized mean difference (SMD) was calculated based on the pre- and post-operative assessments within the study, as mentioned above. We used SMD instead of mean difference as the effect measure because different studies measure the outcomes in different ways, making the absolute value of change (i.e., the mean difference) not comparable among studies. The SMDs were then combined across studies with the random-effects model using the R statistical package (Foundation for Statistical Computing, Vienna, Austria) and displayed as forest plots. For the studies with multiple eligible arms, each arm was included in the meta-analysis as an independent sub-study. The heterogeneity among studies was assessed using the I^2^ statistics, with an I^2^ ≥ 50% indicating significant heterogeneity.

We performed a subgroup analysis by surgery classification, including osteotomy with high and low risks of first metatarsal elevation, arthrodesis (fusion), and soft tissue procedures. The risk of first metatarsal elevation associated with osteotomy, which could affect the plantar load of the first and second metatarsal [30], was determined by an orthopedic surgeon in this review based on the design of the surgical procedure. Other subgroup analyses included year of publication (classified as <2010 vs. ≥2010), median of the reported mean ages of patients (<53 vs. ≥53 years), and median follow-up period (<12 vs. ≥ 12 months). Sensitivity analysis was conducted by removing lower-quality studies with a quality rating of less than half and comparing the meta-analyses results before and after removing the lower-quality studies. Statistical significance level (2-tailed) was set at *p* ≤ 0.05.

## 3. Results

### 3.1. Literature Search and Study Selection

**Figure 2:**
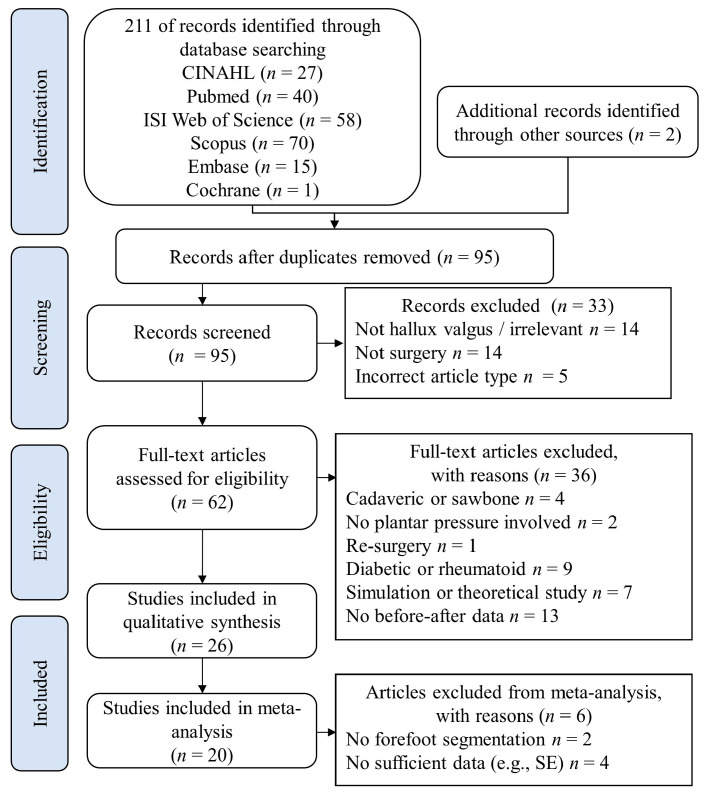
Preferred Reported Items for Systematic Reviews and Meta-Analyses (PRISMA) flowchart for systematic review. CINAHL: Cumulated Index to Nursing and Allied Health Literature; SE: Standard Error.

The literature search identified 211 references, and 2 additional records were found through other sources. A total of 95 references remained after removing duplicates from the initial search. After a preliminary screening of the title and abstract, 33 records were excluded because they were irrelevant to HV (*n* = 14), irrelevant to surgery (*n* = 14), or ineligible article type (e.g., review article, commentary article) (*n* = 5). Sixty-two articles were subject to full-text screening, and thirty-six of them were discarded with the following reasons: cadaveric or sawbone research (*n* = 4), no plantar pressure measurement involved (*n* = 2), re-surgery research (*n* = 1), involving diabetic or rheumatoid foot (*n* = 9), simulation or theoretical research (*n* = 7), and no data on before-after change (*n* = 13). Finally, 26 studies were included in this systematic review after screening [24,25,31,32,33,34,35,36,37,38,39,40,41,42,43,44,45,46,47,48,49,50,51,52,53,54], as shown in Figure 2. Seventeen studies contributed one eligible arm, while nine studies contributed two eligible arms, giving a total of thirty-five arms included in our analysis.

### 3.2. Basic Characteristics of Included Studies

As shown in Table 1, a total of 857 HV patients who underwent surgery (90.7% female, 6.1% male, and 3.3% gender unspecified) were included, involving 973 feet (assuming unilateral cases if the number of feet is not specified). The sample sizes of studies ranged from 4 to 79. Ten studies included female participants only. The age of participants ranged from 16 to 79 years (median of mean age: 51 years). The severity of HV was classified by the Manchester scale [55,56], using the HV angle (HVA) and the first-second intermetatarsal angle (IMA). Severe cases were defined by a HVA ≥ 40° or an IMA ≥ 18°. Moderate cases were defined by a HVA from 21° to 39° or an IMA from 12° to 17°. Mild cases were defined by a HVA from 15° to 20° or an IMA from 9° to 11°. Eleven studies involved cases with severe deformity, eight studies included mild cases, while the others provided no information on severity.

As shown in Table 2, 20 of the 26 studies (77%) investigated the performance of osteotomy procedures involving 28 arms. Twenty-two arms received distal osteotomies, while the other six received proximal osteotomies. Five studies investigated joint fusion, such as the Lapidus procedure, while three studies primarily targeted soft tissue techniques as the primary procedure. Two studies considered both osteotomy and joint fusion procedures [39,54]. No study involved arthroplasty or joint replacement. Two studies involved adjunctive procedures on the lesser toes [35,39].

### 3.3. Outcomes and Measurements

All studies evaluated the plantar load (either in force (*n* = 11) or in pressure (*n* = 22)) or impulse (either in force time-integral (*n* = 10) or in pressure time-integral (*n* = 6)). Most of the studies (20/26) conducted one postoperative measurement, while six studies attempted to compare multiple follow-up time-points. There were 20 studies with their last follow-up time-point at the twelfth month or later. The average follow-up period ranged from 3 months to 7.9 years (median 12 months).

### 3.4. Methodological Quality

As shown in methodological quality assessment in Table S2, among the 26 articles, 18 (69%) scored more than half (5 or above). Most of the studies secured points for “high overall follow-up rate”, “clear study question”, and “intervention clearly described”, while lost points on “blinding of outcome assessors”. As none of the eligible studies reported a publicly available protocol, we were unable to examine whether the outcomes investigated by them were all reported in the published papers.

### 3.5. Data Synthesis

**Figure 3:**
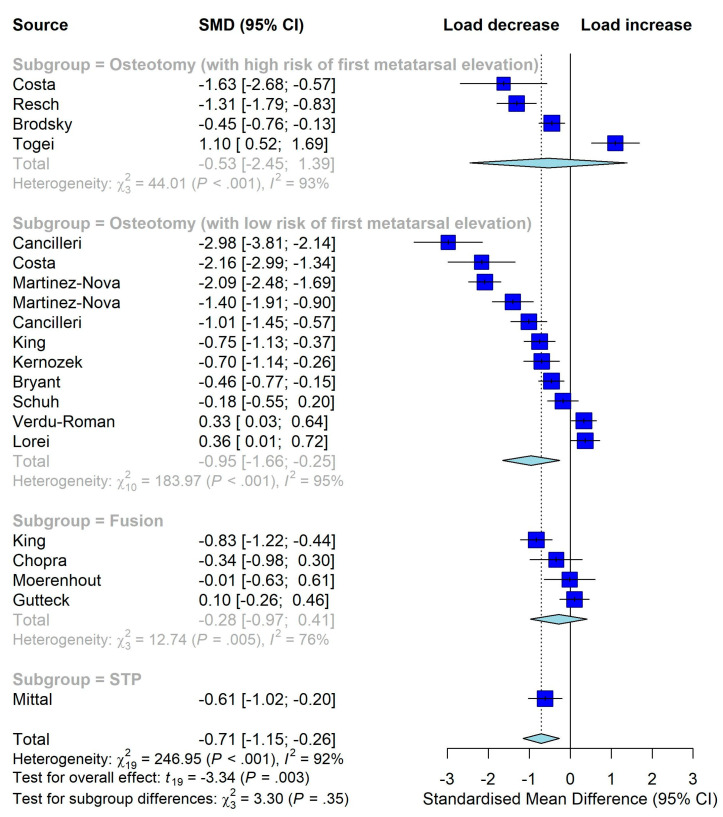
Meta-analysis on the overall effects of surgeries on plantar load over hallux region (increase means better). STP: soft tissue procedure [24,25,32,33,33,34,35,36,38,41,42,43,44,46,49,50,53,54,54].

**Figure 4:**
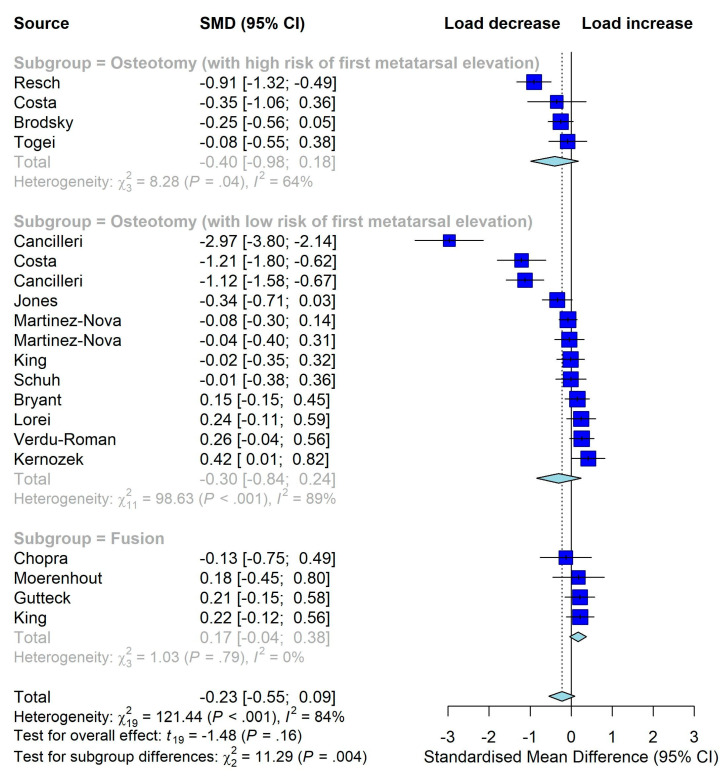
Meta-analysis on the overall effects of surgeries on plantar load over medial metatarsal region (increase means better) [24,25,32,33,34,35,36,37,38,41,42,44,46,49,50,53,54].

**Figure 5:**
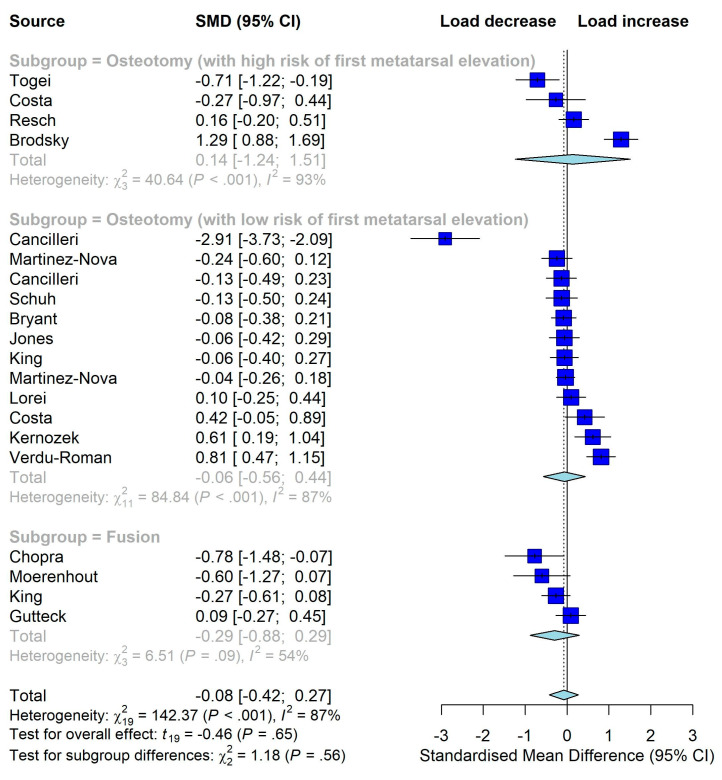
Meta-analysis on the overall effects of surgeries on plantar load over central metatarsal region (decrease means better) [25,32,33,34,35,35,36,37,38,41,42,44,46,49,50,53,54].

Twenty studies were included in the meta-analysis. As shown in Figure 3, HV surgery significantly reduced the plantar load over the hallux (SMD −0.71, 95% CI, −1.15 to −0.26, *p* = 0.003), indicating that surgery did not improve but rather adversely affect push-off function. For the other five outcomes, the overall estimates were not statistically significant, indicating that surgery did not improve them either (Figure 3, Figure 4 and Figure 5). As significant heterogeneity was observed in all meta-analyses, we did not conduct the funnel plot or Egger’s test to assess publication bias since their results could be misleading in the presence of significant heterogeneity [28].

The other six studies were not included for the meta-analysis because neither the standard deviation or variance of the pre-post group difference nor that of the pre and post groups were given. Among them, three reported an improvement in load capacity on the medial forefoot [31,40,51], one reported a worsening load condition [45], while another one reported no significant difference [47]. We could not determine whether the finding was favorable in one study [52].

### 3.6. Subgroup Analyses

Substantial heterogeneity was observed in all meta-analyses. Subgroup analyses showed that, after surgeries, the impulse over the central metatarsal region was increased (meaning “worsened”) within a follow-up period of ≤12 months (SMD 0.54, 95% CI, 0.12 to 0.95, *p* = 0.031) but not much changed after 12 months (meaning “not improved”) (*p* for subgroup difference = 0.008). The subgroups defined by the classification of surgeries on the load over the medial metatarsal region were quantitatively different (*p* for subgroup difference = 0.004), but there was insufficient evidence that the change in load within any of the subgroups was statistically significant. None of the other subgroup analyses, including those by year of publication, showed a statistically significant subgroup difference, which indicates that the substantial heterogeneity, in most cases, could not be explained by pre-planned subgroup analyses (Table 3 and Table 4). Forest plots of impulses are shown in Figure S1–S3.

### 3.7. Sensitivity Analyses

Sensitivity analyses demonstrated that the impulse over the central metatarsal was significantly increased (SMD 0.27, 95% CI, 0 to 0.53, *p* = 0.05, meaning “worsened”) after removing studies with lower quality scores. The results of the other sensitivity analyses were consistent with those of the main analyses.

## 4. Discussion

Our meta-analyses demonstrated a reduction in hallux and medial forefoot load/impulse that implicated the failure of surgeries to restore forefoot functions. Besides, the pain-causing load at the central forefoot was not lessened. The pathomechanics that manifested transfer metatarsalgia was thus not resolved.

Although substantial heterogeneity was observed in all the meta-analyses, most of the individual studies did not favor surgeries, and some did admit that surgical interventions failed to restore normative plantar functions or produced no significant biomechanical improvement [25,33,35,38,39,54]. One possible explanation for the finding is that some of the included studies were old, and the surgical technique or plantar pressure instrument might have been flawed at that time. Nevertheless, subgroup analyses by year of publication indicated that this lack of effectiveness was unlikely due to whether the surgical methods were old or relatively new. Another reason for the failure could be premature ambulation with pain, stiffness, and weakened intrinsic muscles [35,54]. Indeed, a significantly worse load distribution on the hallux and central metatarsal region was observed in the studies with a shorter follow-up period (<12 months). A third reason could be related to the elevation of first metatarsal head in the surgical procedure in some studies, which might produce negative impact on the plantar pressure. Elevated or a more dorsal position of the first metatarsal head might reduce the load-carrying capacity of the first ray, which was recognized as the cause of metatarsalgia and poor surgical outcomes [30]. Some osteotomy techniques, such as Crescentic [25], closing wedge [57], and Weil [58] are vulnerable to the elevation of the first metatarsal. Nevertheless, our subgroup analysis did not support this fact as the source of heterogeneity. A fourth reason is that the surgeries may not correct or ameliorate hypermobility or instability of the forefoot, which is the etiology of HV [59]. Besides, mainstream surgical techniques may fail to repair the stabilizing soft tissue structures and the underlying soft tissue deficiency or imbalance, which adversely affect the load-carrying capacity [60].

The “negative” biomechanical effects of HV surgeries demonstrated by this systematic review seem to contradict the positive clinical improvement after surgeries. We hypothesize that immediate pain relief and restoration of daily functions might not necessarily complement the resumption of normal foot kinematics and walking capability, which is a secondary measure to contemplate potential deformity recurrence, complications, compensatory foot problems, and falling risks. Surgeries treat the bone misalignment of HV but might not be treating the root cause of the problem.

The findings of this study should not be interpreted as a denial of the merits of HV surgeries. In fact, HV surgeries could remedy bunion (swollen joint) problems, shoe-fitting issues, and facilitate immediate pain relief. Moreover, the restoration of bone alignment ameliorates push-off functions by correcting the position of the sesamoid bones and muscle directions. Radiographic assessment and patient-reported outcomes are undoubtedly the primary outcomes, reflecting deformity correction and immediate pain relief. Yet, these evaluation measures are insufficient, and perceived pain relief may not necessarily be associated with restoration of biomechanical functions [47]. Plantar load measurements examine whether the corrected foot could resume normal foot kinematics and walking capability and could serve as a secondary measure to contemplate potential deformity recurrence, complications, compensatory foot problems, and falling risks. Thus, it would be desirable to have surgeries that are effective in improving plantar load distribution. Some surgeons endeavor to develop alternative surgical methods, including metatarsal suturing techniques (e.g., mini-tightrope) that could reinforce the site stability and minimize the risk of traumatizing osseous procedures [61]. However, the effectiveness of these methods warrants further investigation. Some current osteotomies have taken care of the plantar pressure changes during the repositioning and fixation but were not covered in this review due to a lack of pressure testing data.

Postoperative rehabilitation, such as orthosis and muscle training, plays an important role in load redistribution and regaining foot functions. Postoperative muscle retraining could strengthen hallux functions, restore joint mobility and thus physiological gait patterns [49,62]. Schuh et al. [49] commented that the strengthening of peroneus longus muscle can facilitate a better midfoot pronation control and therefore direct load to the first ray correctly. Foot orthosis with arch support could also help control pronation [63], while a metatarsal pad could relieve pain and maintain the integrity of the transverse arch, in cases of first ray insufficiency [64,65,66]. Besides, despite that an increase (or a restoration) of first ray load indicated the restoration of biomechanical functions, it should be noted that unloading the first ray by immobilization or partial weight bearing in the early postoperative stage is essential to facilitate pain management and mitigate risks of non-union [44,50,67].

The substantial heterogeneity observed in our meta-analyses could be caused by clinical and/or methodological factors. For example, HV might induce different types of toe deformities [68]. Patients with lesser toe deformities had an increased peak pressure under the toes or at the tips of the toes but impaired load-carrying capacity due to the reduced contact area of the toes, in addition to the risks of metatarsalgia [69]. Some studies included in this systematic review involved adjunctive procedures of the lesser toes but did not analyze them separately. Furthermore, surgeons might have different preferences on levels of tendon/ligament/capsular release, resection of medial eminence, and adjunctive procedures such as tendon transposition and gastrocnemius elongation.

Besides the variations of surgical techniques, the intrinsic features of HV, such as spring ligament insufficiency [70], first ray hypermobility [71], hypermobility due to malpractice in amateur ballet dancers [72], generalized ligament laxity [73], medial column instability, and posterior tibial tendon dysfunction [74], may have contributed to clinical heterogeneity as well. Moreover, HV was often compounded with other foot problems that were infeasible to isolate [59,75], such as flatfoot [76], plantar fasciitis [77], transfer metatarsalgia [18], and claw toes [78]. There is limited research on the impact of plantar pressure under such circumstances. Those additional foot problems may contribute to variations in plantar loading pattern or postoperative compensatory gait [18,79]. For example, individuals with flat feet might not have sufficient load under the medial forefoot during push-off [80], while those with valgus hindfoot deformities might have higher medial forefoot pressures [81].

As shown by our subgroup analyses, the follow-up period could be one source of the methodological heterogeneity. Studies with multiple postoperative time-point assessments demonstrated a V-shape trend of the plantar pressure during the first year. A second potential source is the approach to measuring plantar pressure. Plantar pressure measurement walkway could address the biomechanics under barefoot conditions, while in-shoe plantar pressure instruments are susceptible to confounding caused by footwear conditions. A previous study commented that the measurement systems themselves might have an effect on the measurement outcomes and thus recommended against using them interchangeably [82]. Walking speed and step width may also affect the plantar pressure measurements [83,84]. The choice of outcome variable for load and impulse (e.g., force or pressure, the mean, or the peak) may have contributed to the observed heterogeneity as well.

This study has some limitations. First, there was substantial heterogeneity among studies, which in most cases could not be resolved by the subgroup analysis. However, as mentioned above, the results of most individual studies did not favor surgeries, suggesting that the heterogeneity does not influence our overall findings. Second, HV compounded with other foot deformities and/or HV surgeries with adjunctive procedures might affect the plantar load but could not be analyzed in an isolated manner in this systematic review, because no primary studies reported related data separately. Third, six studies provided no detailed data that were required for meta-analysis, but their findings also conflicted with each other, which was similar to the situation of other studies. Thus, it is unlikely that the detailed data of these studies, if available and included in our meta-analyses, would influence the main findings notably. Fourth, the evaluation of small study effects was not conducted because of the high heterogeneity in the meta-analysis. Lastly, there were other biomechanical parameters, such as the trajectory of the center of pressure and the contact area, that could reflect the foot’s functions but were not included in the scope of our review.

## 5. Conclusions

While HV surgeries had previously demonstrated improvement in shoe fitting, pain relief on the bunion, and quality of living, there was no solid evidence that HV surgeries could improve forefoot functions from a biomechanical point perspective. Currently available evidence even suggests that surgeries might reduce the plantar load over the hallux and adversely affect push-off function.

## Figures and Tables

**Table 1:** Patient information of the reviewed articles.

Source	Year	Intervention/Group	Subject	Feet	Age *	Deformity Angle	Severity
Borton and Stephens [31]	1994	Basal Chevron ost.	31 (25 F/6 M)	32	53.1	IMA > 12°	Moderate to severe #
Brodsky, et al. [25]	2006	Mod. McBride proc. w/ prox. crescentic ost.	32 (29 F/3 M)	43	41.7 (10.1)	-	-
Bryant, et al. [32]	2005	Austin bunionectomy	31 (27 F/4 M)	44	50.5 (11.3)	HVA ≥ 20°	Moderate to severe #
Cancilleri, et al. [33]	2008	Austin ost.	30 F	30	56.2	IMA < 15°	Mild
		Boc ost.	30 F	30	59.1		
Chopra, et al. [34]	2016	Mod. Lapidus proc.	10 F	-	51.3 (10.3)	-	Moderate to severe
Costa, et al. [35]	2010	Mod. distal Chevron ost.	12 F	19	49 (13)	HVA: 17°–44° IMA: 11°–18°	Mild to moderate
		Mod. distal Chevron ost. w/ Weil proc.	4 F	8			
Gutteck, et al. [36]	2018	1st TMT arthrodesis	28	30	52.5	HVA: 35.9° (8.3°) IMA: 19.2° (3.2°)	Moderate to severe #
Jones, et al. [37]	2004	Scarf ost. w/ Akin closing-wedge ost.	24 (21 F/3 M)	35	46	HVA: 24°–46°IMA: 10°–19°	Moderate to severe #
Kernozek and Sterriker [38]	2002	Chevron (Austin) ost.	25 F	-	43	HVA: 31.7° (4.7°) IMA: 14.5° (1.7°)	Mild to moderate
King, et al. [54]	2014	Chevron bunionectomy	34 (30 F/4 M)	34	55.6 (11.8)	HVA: 24.4° (3.8°) IMA: 13.6° (2.9°)	Moderate #
		Lapidus arthrodesis	34 (32 F/2 M)	34	52.6 (12.0)	HVA: 31.6° (7.0°) IMA: 15.6 ° (4.2°)	
Klemola, et al. [39]	2017	Chevron ost.	30 F	30	37.6 (7.0)	HVA ≤ 50° IMA ≤ 21°	Mild to severe #
		1st TMT arthrodesis	30 (29 F/1 M)	30	51.3 (9.2)	Matched pair on HVA with Chevron group	
Lipscombe, et al. [40]	2008	Scarf ost.	22 (20 F/2 M)	31	57	HVA: 20°–40° IMA: 11°–18°	Moderate #
Lorei, et al. [24]	2006	Scarf ost.	32 (31 F/1 M)	32	54.1 (12.3)	HVA: 32.5° (7.2°) IMA: 15.5° (2.7°)	Moderate #
Martínez-Nova, et al. [42]	2008	PDSTR—Akin proc.	26 F	30	50.3	HVA: 15°–30° IMA ≤ 13°	Mild
Martinez-Nova, et al. [41]	2011	PDSTR—Akin proc.	79 F	79	54.7 (12.5)	HVA: 15°–30°IMA ≤ 13°	Mild
Mittal, et al. [43]	2006	Mod. McBride proc.	19 F	27	49.7	HVA ≥ 20° IMA ≥ 10°	Moderate to severe #
Moerenhout, et al. [44]	2019	Mod. Lapidus proc.	10 F	-	51.3 (8.2)	HVA > 20° IMA > 15°	Moderate to severe
Nyska, et al. [45]	1998	Distal soft tissue proc. w/ prox. ost.	17 (15 F/2 M)	29	47.8	HVA: 29.6° (10.1°) IMA: 12.9° (4.3°)	Moderate #
		Scarf ost.	25 (23 F/2 M)		51.0		
Resch and Stenström [46]	1995	Chevron ost.	24 (23 F/1 M)	22	52	HVA: 32° (8°) IMA: 13° (3°)	Moderate #
		Proximal closing wedge ost.		9		HVA: 31° (7°) IMA: 12° (3°)	
Saro, et al. [47]	2007	Chevron ost.	8 F	8	49 (13)	HVA: 20°–44° IMA < 21°	Moderate to severe #
		Lindgren ost.	14 F	14	49 (14)		
Schuh, et al. [49]	2009	Austin ost.	30 (28 F/2 M)	-	58.4 (13.8)	IMA < 16°	Mild to moderate
		Scarf ost.		-		IMA > 16°	
Schuh, et al. [48]	2010	Chevron ost.	29 (28 F/1 M)	-	58	HVA: 20°–50° IMA: 11°–18°	Mild to moderate
Togei, et al. [50]	2020	1st MT prox. crescentic ost. w/ lesser MT prox. shortening ost.	18 F	18	60.4 (7.2)	HVA > 25° IMA > 12°	Moderate to severe
Verdu-Roman, et al. [53]	2020	Mod. Chevron ost.	44 (35 F/9 M)	-	56.1 (12.7)	HVA: 21°–40°	Moderate
Wong, et al. [51]	2014	Syndesmosis	27 (26 F/1 M)	54	46	HVA: 24.3°–49.8° IMA: 10.2°–18.6°	Moderate to severe
Yildiz, et al. [52]	2021	Distal Chevron ost.	26 (22 F/4 M)	-	45.3 (15.2)	HVA: 31.4° (3.9°) IMA: 12.3° (2.6°)	Moderate #
		Proximal Dome ost.	22 (18 F/4 M)	-	44.7 (15.1)	HVA: 38.5° (7.6°) IMA: 14.7° (3.7°)	

* Age is shown as mean (standard deviation). -: not available. # The study provided no information on severity of the deformity. The note in this table was made using the Manchester scale primarily based on HVA [55,56]. F: females; HVA: hallux valgus angle; IMA: first-second intermetatarsal angle; IC.: Inclusion criteria; mod.: modified; M: Males; MT: metatarsal; ost.: osteotomy; proc.: procedure; prox.: proximal; PDSTR: percutaneous distal soft tissue release; TMT: tarsometatarsal; w/: with.

**Table 2:** Information of surgical interventions of the reviewed articles.

Source	Intervention/Group	Surgery Class	Last F/U (Months)	Outcome
Borton and Stephens [31]	Basal Chevron ost.	Osteotomy (proximal)	9.9	MP
Brodsky, et al. [25]	Mod. McBride proc. w/ prox. crescentic ost.	Osteotomy (proximal)	29	PP, PTI
Bryant, et al. [32]	Austin bunionectomy	Osteotomy (distal)	24	PP
Cancilleri, et al. [33]	Austin ost.	Osteotomy (distal)	24	PP, PTI
	Boc ost.	Osteotomy (distal)		
Chopra, et al. [34]	Mod. Lapidus proc.	Fusion	6	PF, PP
Costa, et al. [35]	Mod. distal Chevron ost.	Osteotomy (distal)	3	PP, PTI
	Mod. distal Chevron ost. w/ Weil proc.	Osteotomy (distal)		
Gutteck, et al. [36]	1^st^ TMT arthrodesis	Fusion	12	PF, FTI
Jones, et al. [37]	Scarf ost. w/ Akin closing-wedge ost.	Osteotomy (distal)	12	PP
Kernozek and Sterriker [38]	Chevron (Austin) ost.	Osteotomy (distal)	12	PF, FTI, PP, PTI
King, et al. [54]	Chevron bunionectomy	Osteotomy (distal)	7.7	MP
	Lapidus arthrodesis	Fusion		
Klemola, et al. [39]	Chevron ost.	Osteotomy (distal)	7.9 years	FTI
	1^st^ TMT arthrodesis	Fusion	5.1 years	
Lipscombe, et al. [40]	Scarf ost.	Osteotomy (distal)	12	PP, PTI, FTI
Lorei, et al. [24]	Scarf ost.	Osteotomy (distal)	33	PF, PP, FTI
Martínez-Nova, et al. [42]	PDSTR—Akin proc.	Osteotomy (distal)	12.1	PP, MP
Martinez-Nova, et al. [41]	PDSTR—Akin proc.	Osteotomy (distal)	28.1	MP
Mittal, et al. [43]	Mod. McBride proc.	Soft Tissue proc.	7	PF, PP
Moerenhout, et al. [44]	Mod. Lapidus proc.	Fusion	12	PF, PP
Nyska, et al. [45]	Distal soft tissue proc. w/ prox. ost.	Osteotomy (proximal)	18.2	PF, PP, FTI, PTI
	Scarf ost.	Osteotomy (distal)		
Resch and Stenström [46]	Chevron ost.	Osteotomy (distal)	25	PP
	Proximal closing wedge ost.	Osteotomy (proximal)		
Saro, et al. [47]	Chevron ost.	Osteotomy (distal)	12	PP, MP
	Lindgren ost.	Osteotomy (distal)		
Schuh, et al. [49]	Austin ost.	Osteotomy (distal)	6	PF, PP, FTI
	Scarf ost.	Osteotomy (distal)		
Schuh, et al. [48]	Chevron ost.	Osteotomy (distal)	12	PF, FTI
Togei, et al. [50]	1st MT prox. crescentic ost. w/ lesser MT prox. shortening ost.	Osteotomy (proximal)	18.7	PF, PP, FTI
Verdu-Roman, et al. [53]	Mod. Chevron ost.	Osteotomy (distal)	12	PP, MP
Wong, et al. [51]	Syndesmosis	Soft Tissue proc.	26.4	PF, FTI
Yildiz, et al. [52]	Distal Chevron ost.	Osteotomy (distal)	12	MP
	Proximal Dome ost.	Osteotomy (proximal)		

FTI: force-time integral; F/U: follow-up; PF: peak force; MP: mean pressure; MT: metatarsal; ost.: osteotomy; proc.: procedure; prox.: proximal; PP: peak pressure; PTI: pressure-time integral; PDSTR: percutaneous distal soft tissue release; TMT: tarsometatarsal; w/: with.

**Table 3:** Subgroup and sensitivity analysis of the meta-analysis on load parameters.

	Load over Hx		Load over MMT		Load over CMT	
	Effect (95%CI)	I^2^(%)	Effect (95%CI)	I^2^(%)	Effect (95%CI)	I^2^(%)
Subgroup: Surgery						
Osteotomy (high-elev.)	−0.53 (−2.45 to 1.39)	93	−0.40 (−0.98 to 0.18)	64	0.14 (−1.24 to 1.51)	93
Osteotomy (low-elev.)	−0.95 (−1.66 to −0.25) *	95	−0.30 (−0.84 to 0.24)	89	−0.06 (−0.56 to 0.44)	87
Fusion	−0.28 (−0.97 to 0.41)	76	0.17 (−0.04 to 0.38)	0	−0.29 (−0.88 to 0.29)	54
STP	−0.61 (01.02 to −0.20)	-	-		-	
SGD	*p* = 0.35		*p* = 0.004		*p* = 0.56	
Subgroup: Age						
<53	−0.75 (−1.15 to −0.34) *	81	−0.15 (−0.45 to 0.15)	76	0.06 (−0.29 to 0.40)	82
>=53	−0.63 (−1.74 to −0.49) *	96	−0.37 (−1.19 to 0.45)	91	−0.29 (−1.12 to 0.55)	91
SGD	*p* = 0.81		*p* = 0.55		*p* = 0.38	
Subgroup: Follow-up period						
>=12 months	−0.63 (−1.28 to 0.02)	95	−0.24 (−0.69 to 0.21)	87	−0.06 (−0.56 to 0.45)	90
<12 months	−0.81 (−1.40 to −0.21) *	75	−0.2 (−0.71 to 0.32)	72	−0.13 (−0.48 to 0.23)	47
SGD	*p* = 0.64		*p* = 0.88		*p* = 0.79	
Subgroup: Publication Year						
<2010	−0.81 (−1.41 to −0.20) *	90	−0.41 (−1.06 to 0.24)	90	−0.07 (−0.77 to 0.63)	91
>=2010	−0.60 (−1.36 to 0.17)	94	−0.04 (−0.32 to 0.24)	62	−0.09 (−0.44 to 0.27)	79
SGD	*p* = 0.63		*p* = 0.24		*p* = 0.96	
Sensitivity Analysis						
Overall	−0.71 (−1.15 to −0.26) *	92	−0.23 (−0.55 to 0.09)	84	−0.08 (−0.42 to 0.27)	87
RL	−0.71 (−1.17 to −0.25) *	92	0.00 (−0.21 to 0.20)	60	0.00 (−0.23 to 0.23)	72

CMT: central metatarsal; elev.: elevation; Hx: hallux; MMT: medial metatarsal; RL: remove low quality articles; SGD: subgroup difference; STP: soft tissue procedure; high-elev.: with high risk of first metatarsal elevation; low-elev.: with low risk of first metatarsal elevation; * significant effect (*p* < = 0.05)

**Table 4:** Subgroup and sensitivity analysis of the meta-analysis on impulse parameters.

	Impulse over Hx		Impulse over MMT		Impulse over CMT	
	Effect (95%CI)	I^2^(%)	Effect (95%CI)	I^2^(%)	Effect (95%CI)	I^2^(%)
Subgroup: Surgery						
Osteotomy (high-elev.)	−0.39 (−6.01 to 5.22)	94	−0.05 (−0.38 to 0.29)	0	−0.04 (−0.78 to 0.70)	37
Osteotomy (low-elev.)	−0.70 (−1.68 to 0.27)	95	−0.05 (−0.68 to 0.58)	91	0.02 (−0.58 to 0.61)	89
Fusion	−0.22 (−2.48 to 2.04)	45	0.25 (−3.97 to 4.48)	84	−0.08 (−1.04 to 0.89)	0
STP	-		-		-	
SGD	*p* = 0.55		*p* = 0.68		*p* = 0.93	
Subgroup: Age						
<53	−0.80 (−1.63 to 0.02)	85	0.01 (−0.44 to 0.46)	76	0.07 (−0.18 to 0.32)	41
>=53	−0.07 (−1.57 to 1.42)	96	0.03 (−0.78 to 0.84)	92	−0.13 (−0.97 to 0.71)	91
SGD	*p* = 0.28		*p* = 0.96		*p* = 0.57	
Subgroup: Follow-up period						
>=12 months	−0.33 (−1.09 to 0.44)	93	0.02 (−0.37 to 0.40)	85	−0.13 (−0.53 to 0.26)	83
<12 months	−1.50 (−6.06 to 3.05)	95	0.00 (−2.38 to 2.39)	93	0.54 (0.12 to 0.95)*	0
SGD	*p* = 0.29		*p* = 0.98		*p* = 0.008	
Subgroup: Publication Year						
<2010	−0.64 (−1.78 to 0.49)	95	−0.04 (−0.82 to 0.74)	92	−0.18 (−0.96 to 0.59)	90
>=2010	−0.40 (−1.74 to 0.94)	93	0.07 (−0.40 to 0.55)	77	0.14 (−0.17 to 0.46)	58
SGD	*p* = 0.73		*p* = 0.75		*p* = 0.32	
Sensitivity Analysis						
Overall	−0.51 (−1.26 to 0.23)	93	0.02 (−0.35 to 0.38)	87	0 (−0.34 to 0.33)	82
RL	−0.47 (−1.48 to 0.54)	92	0.19 (−0.26 to 0.65)	80	0.27 (0 to 0.53) *	56

CMT: central metatarsal; elev.: elevation; Hx: hallux; MMT: medial metatarsal; RL: remove low quality articles; SGD: subgroup difference; STP: soft tissue procedure; high-elev.: with high risk of first metatarsal elevation; low-elev.: with low risk of first metatarsal elevation; * significant effect (*p* < = 0.05).

## Data Availability

Data sharing is not applicable to this review article.

## Supplementary Materials

The following supporting information can be downloaded at: https://www.mdpi.com/article/10.3390/jcm12041384/s1, Figure S1: Meta-analysis on the overall effects of surgeries on plantar impulse (load time-integral) over hallux region (increase means better); Figure S2: Meta-analysis on the overall effects of surgeries on plantar impulse (load time-integral) over medial metatarsal region (increase means better); Figure S3: Meta-analysis on the overall effects of surgeries on plantar impulse (load time-integral) over central metatarsal region (decreases means better). Table S1: Search terms and strategy in the systematic review; Table S2: Methodological quality assessment using NIH quality assessment tool for before-after study.
