# Supplementary material for: Forefoot Function after Hallux Valgus Surgery: A Systematic Review and Meta-Analysis on Plantar Load Measurement
Source: J Clin Med. 2023 Feb 9;12(4):1384. doi: 10.3390/jcm12041384 (PMC9965975; doi:10.3390/jcm12041384)
Supplement: Supplementary file 1 [file jcm-12-01384-s001.zip › Table S1 Search Strategy.pdf]

Table S1. Search Strategies per database

Database	Field	Filter	Search Terms
Web of Science	Title, abstract, keywords, keywords plus	Type: Article<br>Language: English	"plantar load" OR "plantar pressure" OR pedobarograph* OR barograph* OR baropodometr*<br><br>AND<br><br>bunion OR "hallux valgus" OR "hallux abducto valgus" OR "hallux abductovalgus" OR "hallux varus"<br><br>AND<br><br>osteotom* OR arthrodesis OR fusion OR surgery OR surgical OR "tissue procedure" OR arthroscop* OR implant OR arthroplasty OR osteodesis OR syndesmosis OR suture OR tightrope
Scopus	Title, abstract, keywords,	Type: Article<br>Source: Journal<br>Language: English	
Ovid (Search All Ovid Journals)	Title, abstract, keywords,	Type: Article	
CINAHL Complete via EBSCOHost	Not specified (i.e. Select a field [optional])	Type: Research Article<br>Language: English, Apply related words<br>Apply equivalent subjects	
CENTRAL, Cochrane	Title, Abstract, keywords	N/A	"plantar load" OR "plantar pressure" OR pedobarograph* OR barograph* OR baropodometr*<br><br>AND<br><br>Bunion [MeSH Terms] OR hallux valgus [MeSH Terms]<br><br>AND<br><br>osteotom* OR arthrodesis OR fusion OR surgery OR surgical OR "tissue procedure" OR arthroscop* OR implant OR arthroplasty OR osteodesis OR syndesmosis OR suture OR tightrope
Pubmed	Title, Abstract	Type: Journal Article<br>Language: English	
