# Supplementary material for: Forefoot Function after Hallux Valgus Surgery: A Systematic Review and Meta-Analysis on Plantar Load Measurement
Source: J Clin Med. 2023 Feb 9;12(4):1384. doi: 10.3390/jcm12041384 (PMC9965975; doi:10.3390/jcm12041384)
Supplement: Supplementary file 1 [file jcm-12-01384-s001.zip › Table S2 Method Quality Assessment.pdf]

Table S2. Methodological Quality Assessment using NIH Quality Assessment Tool for Before-after Study

	Study Question	Eligibility	Representativeness	Enrollment Criteria	Intervention	Outcome Measures	Assessor Blinding	Follow-up	Multiple Measure	Sum (out of 9)
Borton and Stephens [32]	0	0	1	0	1	0	0	1	0	3
Brodsky, et al. [26]	1	0	0	0	1	0	0	1	1	4
Bryant, et al. [33]	0	1	0	1	0	1	0	1	1	5
Cancilleri, et al. [34]	1	0	0	1	1	0	0	1	0	4
Chopra, et al. [35]	1	0	1	1	1	1	0	1	1	7
Costa, et al. [36]	1	0	0	1	1	0	0	1	1	5
Gutteck, et al. [37]	0	0	1	1	0	0	0	1	0	3
Jones, et al. [38]	1	0	1	1	1	0	0	1	1	6
Kernozek and Sterriker [39]	1	0	0	1	1	0	0	1	1	5
King, et al. [55]	1	1	1	1	1	0	0	1	1	7
Klemola, et al. [40]	1	1	0	1	1	0	1	1	0	6
Lipscombe, et al. [41]	1	1	1	1	1	0	0	0	1	6
Lorei, et al. [25]	1	1	1	1	1	0	0	1	1	7
Martinez-Nova, et al. [43]	1	1	0	1	1	1	0	1	1	7
Martinez-Nova, et al. [42]	1	0	0	1	1	1	0	1	1	6
Mittal, et al. [44]	1	1	0	1	1	0	0	1	1	6
Moerenhout, et al. [45]	1	1	1	1	0	1	0	1	1	7
Nyska, et al. [46]	1	0	0	0	1	0	0	1	0	3
Resch and Stenström [47]	0	0	1	0	0	0	0	1	1	3
Saro, et al. [48]	1	1	1	1	0	1	0	1	1	7
Schuh, et al. [50]	1	0	1	0	1	1	0	1	1	6
Schuh, et al. [49]	1	0	1	0	1	1	0	1	1	6
Togei, et al. [51]	1	0	0	0	1	0	0	1	1	4
Verdu-Roman, et al. [54]	1	1	1	1	1	1	0	1	1	8
Wong, et al. [52]	0	0	1	0	1	0	0	1	1	4
Yildiz, et al. [53]	0	0	1	1	1	1	1	1	0	6
Sum (26 articles)	20	10	15	18	21	10	2	25	20	
