# Supplementary figures and images for: Forefoot Function after Hallux Valgus Surgery: A Systematic Review and Meta-Analysis on Plantar Load Measurement
Source: J Clin Med. 2023 Feb 9;12(4):1384. doi: 10.3390/jcm12041384 (PMC9965975; doi:10.3390/jcm12041384)

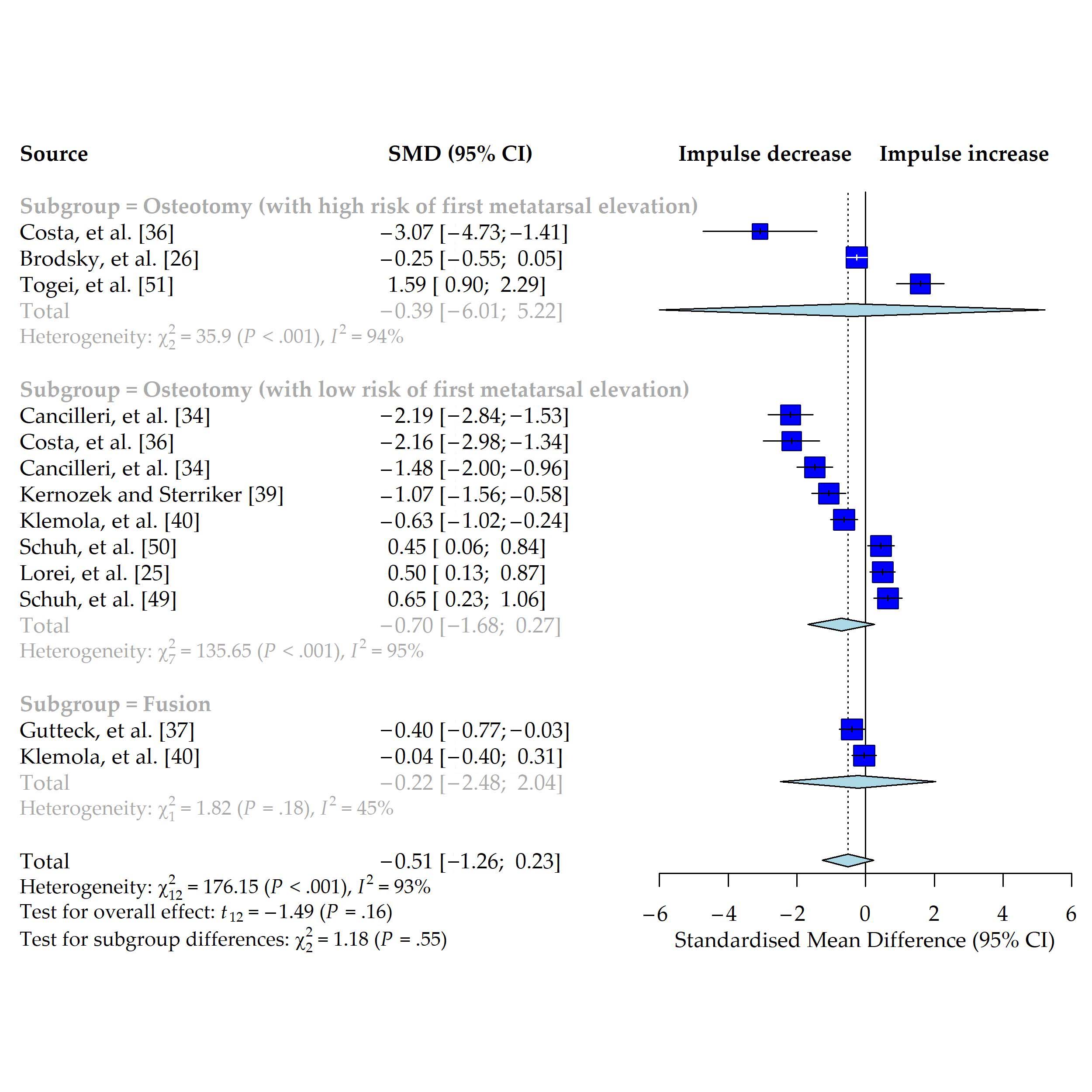

Supplement: Supplementary file 1 [file jcm-12-01384-s001.zip › Figure S1.jpg]

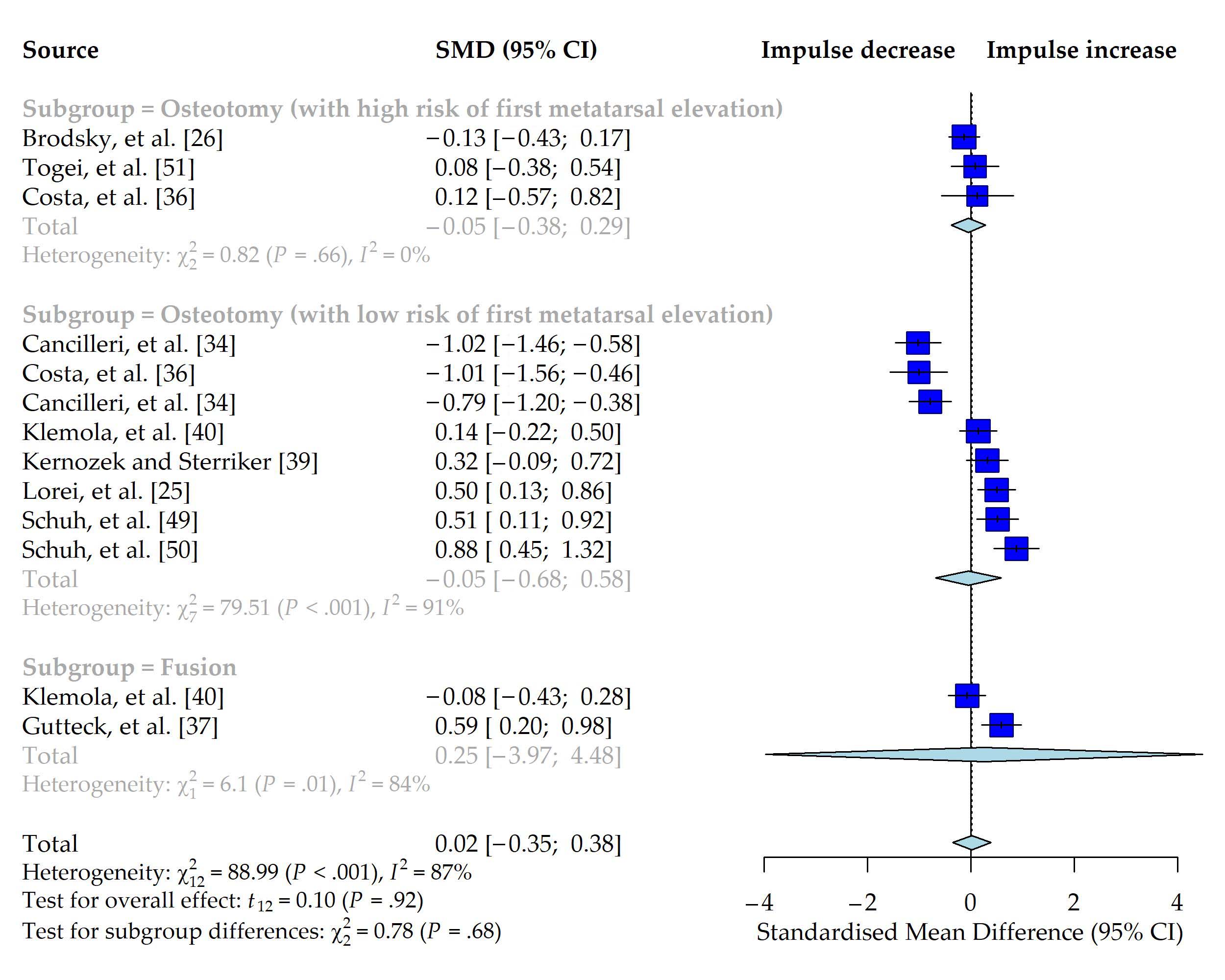

Supplement: Supplementary file 1 [file jcm-12-01384-s001.zip › Figure S2.jpg]

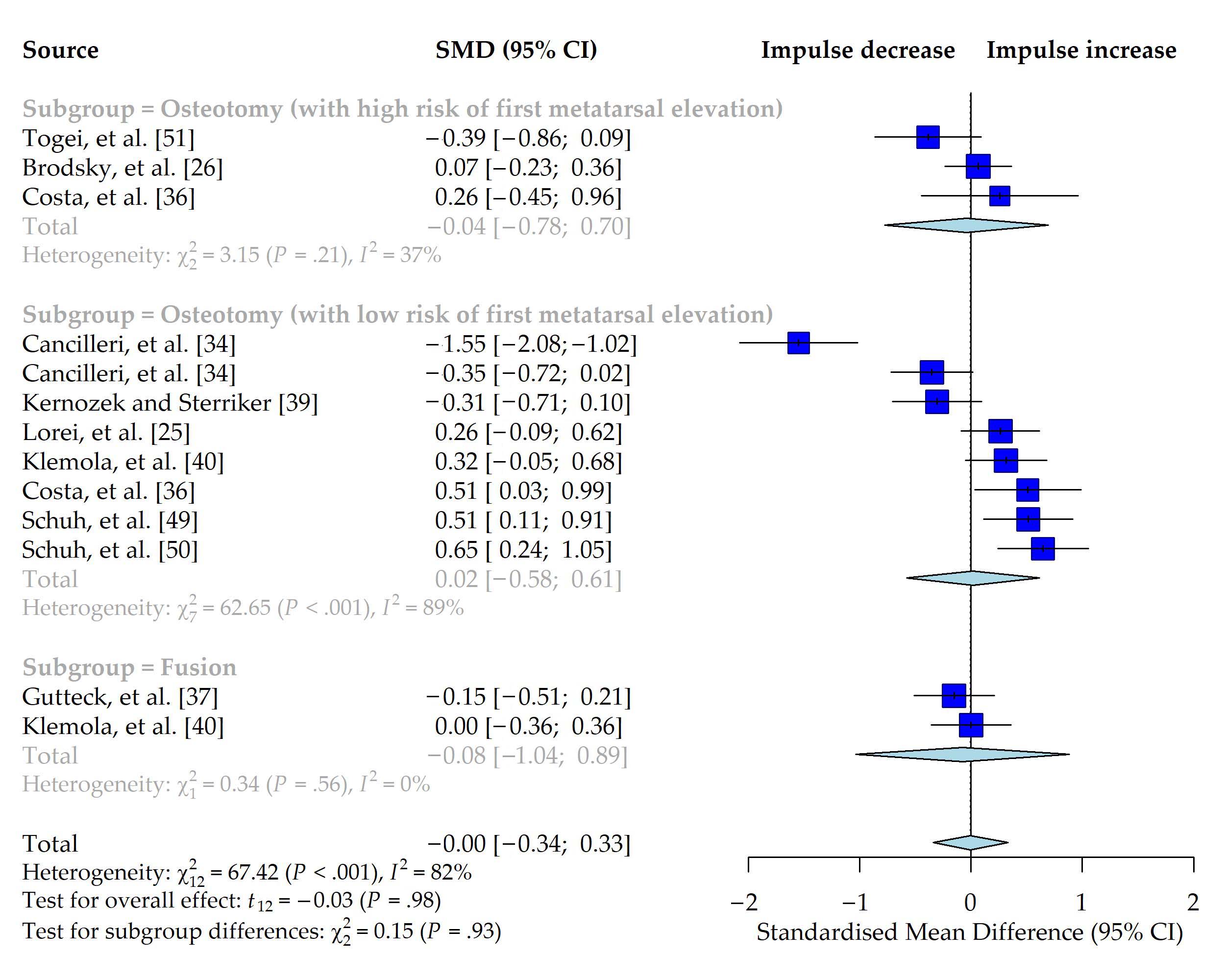

Supplement: Supplementary file 1 [file jcm-12-01384-s001.zip › Figure S3.jpg]
